# Two-Host, Two-Vector Basic Reproduction Ratio (*R*_0_) for Bluetongue

**DOI:** 10.1371/journal.pone.0053128

**Published:** 2013-01-08

**Authors:** Joanne Turner, Roger G. Bowers, Matthew Baylis

**Affiliations:** 1 Department of Epidemiology and Population Health, Institute of Infection and Global Health, University of Liverpool, Leahurst, Neston, United Kingdom; 2 Department of Mathematical Sciences, School of Physical Sciences, University of Liverpool, Liverpool, United Kingdom; Universidad de Zarazoga, Spain

## Abstract

Mathematical formulations for the basic reproduction ratio (*R*_0_) exist for several vector-borne diseases. Generally, these are based on models of one-host, one-vector systems or two-host, one-vector systems. For many vector borne diseases, however, two or more vector species often co-occur and, therefore, there is a need for more complex formulations. Here we derive a two-host, two-vector formulation for the *R*_0_ of bluetongue, a vector-borne infection of ruminants that can have serious economic consequences; since 1998 for example, it has led to the deaths of well over 1 million sheep in Europe alone. We illustrate our results by considering the situation in South Africa, where there are two major hosts (sheep, cattle) and two vector species with differing ecologies and competencies as vectors, for which good data exist. We investigate the effects on *R*_0_ of differences in vector abundance, vector competence and vector host preference between vector species. Our results indicate that *R*_0_ can be underestimated if we assume that there is only one vector transmitting the infection (when there are in fact two or more) and/or vector host preferences are overlooked (unless the preferred host is less beneficial or more abundant). The two-host, one-vector formula provides a good approximation when the level of cross-infection between vector species is very small. As this approaches the level of intraspecies infection, a combination of the two-host, one-vector *R*_0_ for each vector species becomes a better estimate. Otherwise, particularly when the level of cross-infection is high, the two-host, two-vector formula is required for accurate estimation of *R*_0_. Our results are equally relevant to Europe, where at least two vector species, which co-occur in parts of the south, have been implicated in the recent epizootic of bluetongue.

## Introduction

Mathematical formulations for the basic reproduction ratio (*R*_0_) – defined as the average number of secondary infections produced by a typical primary infection in an otherwise totally susceptible population [1] – exist for several vector-borne diseases including those with one host and one vector, such as malaria [2] and those with two hosts and one vector, such as zoonotic trypanosomiasis [3], African horse sickness [4] and bluetongue [5], [6]. To date, with the exception of Lopez et al. [7], almost no attention has been paid to developing mathematical formulations of *R*_0_ where there are both multiple hosts and multiple vectors. However, this is a common situation: for trypanosomiasis in Africa for example, two or more species of tsetse fly vector often co-exist; while for both African horse sickness and bluetongue in southern Africa, two competent vectors (*Culicoides imicola* and *C. bolitinos*) are frequently trapped together. Other diseases transmitted by multiple vectors include dengue [8], Japanese encephalitis [9] and malaria [10].

This situation may also apply to the recent European outbreak of bluetongue, which has caused the deaths of well over a million sheep. The outbreak began in 1998 in regions of southern Europe where the Afrotropical midge, *C. imicola*, occurs. Starting in 1999, it was also detected in Balkan countries where *C. imicola* was not known, thereby implicating local *Culicoides* species, such as the *obsoletus* and *pulicaris* groups, as vectors. Since these co-occur with *C. imicola* over the latter's European range [11], it was reasonable to suspect that they may transmit the virus alongside *C. imicola* in some places; and epidemiological evidence for this was later provided in Sicily [12]. Subsequently, both BTV 1 and BTV 8 have been transmitted in regions with indigenous vectors, both with and without *C. imicola*. It is therefore quite likely that two or more vector species have co-transmitted BT virus in several parts of Europe in recent years.

Given the likely widespread existence of multivector, multihost disease systems, we derive and analyse *R*_0_ for the simplest of these: a two-host, two-vector system, using bluetongue as an example. We particularly wish to investigate the effect on *R*_0_ of measurable parameter values relating to vector abundance, vector competence and vector host preference. In order to do this we extend the work of Lopez et al. [7] by first including vector host preference and temperature-dependent transmission parameters and then studying the effect of specific parameters. We consider the effect of the following: (1) vector to host ratio, which is linked to vector abundance and varies with species and temperature, as evidenced by *C. imicola* and *C. bolitinos* in RSA [13], [14]; (2) probability of transmission from host to vector, which is linked to vector competence and also varies with species and temperature [15]; (3) vector host preference, which differs between species [14], [16], [17]. Importantly, we also work directly with *R*_0_, rather than the threshold *T* proposed by Lopez et al. [7], which is not valid in all regions of feasible parameter space. The notation we use allows us to make direct comparisons with a previously published two-host, one-vector formula for bluetongue. We illustrate our results by parameterising the model for a specific disease system, namely bluetongue in South Africa. We use the situation in South Africa because of the availability of extensive distribution data, together with detailed experimental results on the relative vector competencies of the two main vector species [15]. Similar data for different European bluetongue vectors do not exist. It is known that several European vector species transmit bluetongue virus and that there are differences in host preference between these species. For example, Garros et al. [18] show that *C. chiopterus* prefers to feed on cattle while *C. obsoletus* is more of a generalist. However, nothing is known of their respective vector competencies. This highlights the need for a two-host, two-vector formula for *R*_0_ as well as experimental work to establish the vector competence of each species. Although we have focussed on the situation in South Africa, the framework and general results presented here are equally relevant to Europe.

## Analysis

### Model Equations

Equations describing the dynamics of a two-host, two-vector system are given below, whilst the variables and parameters of the model are defined and described in Table 1. For clarity, we have adopted a similar notation to that used by Gubbins et al. [6]. In short, hosts can be either susceptible, infectious or recovered (and in this case immune), whilst vectors can be either susceptible, latent or infectious. The proportions of susceptible, infectious and recovered hosts are denoted by 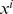, 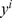 and 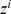 respectively, whilst the numbers of susceptible, latent and infectious vectors are denoted by 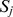, 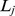 and 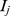 respectively (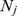 in total). Susceptible hosts of type *i* [where *i* can be either *C* (cattle) or *S* (sheep)] become infectious at rate *λ_Hi_*, which is the sum over vector types indicated by *j* [where *j* can be either 1 (*C. imicola*) or 2 (*C. bolitinos*)] of 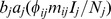. The third term is composed of 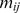 the ratio of vectors of type *j* to hosts of type *i*, 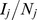 the proportion of vectors of type *j* that are infectious and 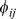 the proportion of vectors of type *j* attracted to hosts of type *i* (i.e. reflecting the preference of vector type *j* for host type *i*). So, the third term gives the average number of infectious vectors of type *j* attracted to a host of type *i* (after taking into account vector type *j*'s preference for host type *i*). This is multiplied by 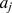, the (temperature-dependent) biting rate of vectors of type *j*, and 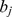, the probability of transmission from a vector of type *j* to a host given an effective contact. Similarly, susceptible vectors of type *j* become latent at rate *λ_Vj_*, which is the sum over host types (indicated by *i*) of 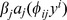. The third term is the probability of a vector of type *j* being attracted to an infectious host of type *i*. This is multiplied by 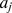, the (temperature-dependent) biting rate of vectors of type *j*, and 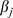, the probability of transmission from a host to a vector of type *j* given an effective contact. An infectious host remains infectious until it either recovers (at rate *r_i_*) or dies (at rate *d_i_*). After a short extrinsic incubation period (on average 1/*ν_j_*), latent vectors become infectious. They remain infectious until they die, which occurs at rate 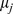. Susceptible vectors are added to the system at rate 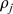. The model assumes that there is no seasonal aspect to vector recruitment or population size and that there is no latent period in hosts, recovered animals are immune and the host population remains constant except for losses due to disease-induced mortality.

**Table 1:** Definitions and descriptions of the variables, parameters and rates that influence the dynamics of the two-host, two-vector system and the parameter values used to estimate *R*_0_.

Variable, Parameter or Rate	Construction	Definition or Description	Point Estimate and/or Feasible Range	Comments and Formula if Temperature-dependent[vector species]
*x^i^*	*X^i^*/*H_i_*	proportion of host type *i* that are susceptible		*i* can be *C* (cattle) or *S* (sheep)
*y^i^*	*Y^i^*/*H_i_*	proportion of host type *i* that are infectious		
*z^i^*	*Z^i^*/*H_i_*	proportion of host type *i* that have recovered		
*H_i_*	*X^i^*+*Y^i^*+*Z^i^*	total number of host type *i*		
*λ_Hi_*	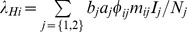	rate at which susceptible hosts of type *i* become infectious through being bitten by infectious vectors		*j* can be 1 (*C. imicola*) or 2 (*C. bolitinos*)
*b_j_*		probability of transmission from vector type *j* to a host given an effective contact	0.8–1.0	[*C. sonorensis*]
*a_j_*		biting rate of vector type *j*	0–0.5	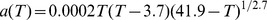 [*C. sonorensis*]
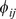	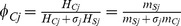 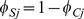	proportion of vectors of type *j* attracted to hosts of type *i*		As Gubbins et al. [6], for clarity we replace 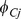 and 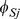 with 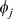 and 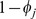 respectively.
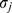		host preference of vector type *j* 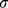 <1 indicates a preference for cattle 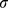 >1 indicates a preference for sheep	0–1	*C. imicola* feeds predominantly on cattle and sheep [16], [17], but prefers cattle [23]. *C. bolitinos* feeds on cattle and horses [16], [17] and breeds in cattle dung [14].
*m_ij_*	*N_j_*/*H_i_*	ratio of vectors of type *j* to hosts of type *i*	Many areas:*m_C_*_1_ = *m_S_*_1_ = 500 (0–5000)*m_C_*_2_ = *m_S_*_2_ = 50 (0–500)Colder high-lying areas:*m_C_*_1_ = *m_S_*_1_ = 50 (0–100)*m_C_*_2_ = *m_S_*_2_ = 500 (0–5000)	In general, *C. imicola* is approx. 10 times more abundant than *C. bolitinos* [15]. In colder, high-lying areas, *C. imicola* is approx. 10 times less abundant than *C. bolitinos* [14].
*N_j_*	*S_j_*+*L_j_*+*I_j_*	total number of vectors of type *j*		
*r_i_*		recovery rate of host type *i*	*r_C_* = 1/20.6*r_S_* = 1/16.4	
*d_i_*		pathogen-induced mortality rate of host type *i*	*d_C_* = 0*d_S_* = 0.001–0.01	
*S_j_*		number of vectors of type *j* that are susceptible		
*L_j_*		number of vectors of type *j* that are latent		
*I_j_*		number of vectors of type *j* that are infectious		
*λ_Vj_*	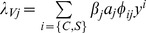	rate at which susceptible vectors of type *j* become latent through biting infectious hosts		
*β_j_*		probability of transmission from a host to vector type *j* given an effective contact	*β*_1_ = 0.0021–0.0654*β*_2_ = 0.0268–0.6444	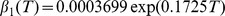 [*C. imicola*] 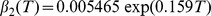 [*C. bolitinos*]Both from data in [15].
*ν_j_*		rate at which latent vectors of type *j* become infectious ( = 1/EIP, where EIP = extrinsic incubation period)	1/4–1/26	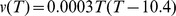 [*C. sonorensis*]
*μ_j_*		natural mortality rate of vector type *j*	0.1–0.5	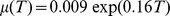 [*C. sonorensis*]
*ρ_j_*		replacement rate of vector type *j*		

Unless otherwise stated, values were taken from Gubbins et al. [6]. Subscripts 1 and 2 denote *C. imicola* and *C. bolitinos* respectively.

#### Hosts



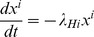


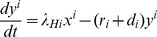


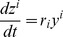
where 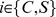.

#### Vectors



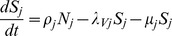


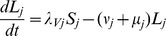


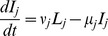
where 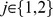.

### Basic Reproduction Ratio

The ability of the pathogen to spread can be expressed in terms of the basic reproduction ratio *R*_0_. Mathematically, *R*_0_ is the dominant eigenvalue of the next-generation matrix *K*. For vector-borne transmission models like the one described above,
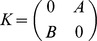
where matrix *A* describes vector to host transmission and matrix *B* describes host to vector transmission (see Appendix 1 in File S1). We could work directly with the characteristic equation 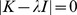. However, there are significant advantages in using a result shown in Appendix 2 in File S1, namely that *R*_0_ is the square root of the dominant eigenvalue of *BA* (a 4×4 submatrix of *K* ^2^). Not only is *BA* smaller than *K* but also its elements have an obvious biological interpretation in terms of *R_ij_*, the average number of infectious vectors of type *i* produced by one infectious vector of type *j* (necessarily in two generations). It is such biological interpretation that we seek. The utility of working with *BA* is doubtless associated with the argument that, in contrast to directly-transmitted infections, for vector-borne infections 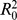 makes more sense biologically [2] and is in fact what is measured in the field (i.e. two-generation ‘like’ to ‘like’ transmission).

Following the above procedure we find that
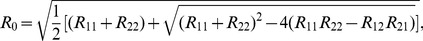
(1)where specifically
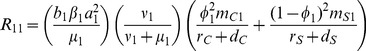
(2)

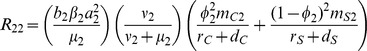


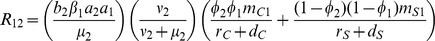


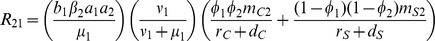
Note that the proportion of vectors of type *j* attracted to hosts of type *i*

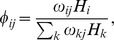
where 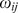 is a measure of vector type *j*'s preference for host type *i* and 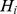 is the total number of hosts of type *i*. For two host species (cattle *C* and sheep *S*), this can be rewritten as
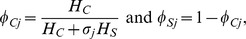
where vector host preference is now denoted by 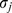. In terms of vector to host ratios, where 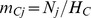 and 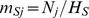, the first of these is
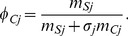
(3)As Gubbins et al. [6], for clarity we replace 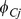 and 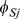 with 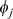 and 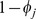 respectively.

From Gubbins et al. [6], we know that 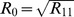 and 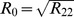 for two-host, one-vector systems involving vector type 1 and vector type 2, respectively. For the two-host, two-vector system we find from equations (2) that when 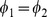, 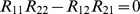 and hence 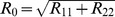. If vectors 1 and 2 are identical in terms of parameter values (i.e. all parameter values for vector species 1 equal those for vector species 2) and equal in number, then 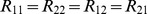 and so 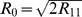. In other words, the two-host, two-vector 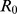 is greater than the two-host, one-vector 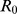 by a factor of 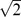. So, in this case, if it were assumed that there was only one vector species transmitting the infection, the basic reproduction ratio would be underestimated (because the average number of relevant vectors per host would be underestimated). When the vectors are not identical, as the level of cross-infection 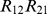 increases, 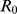 increases from 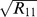 (when 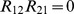 and 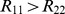), through 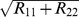 (when 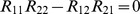) to 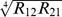 (when 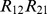 is very large).

## Results

For the two-host, one-vector system, Gubbins et al. [6] found that the parameters with a significant effect on *R*_0_ were temperature (*T*) [via biting rate, extrinsic incubation period (EIP), vector mortality rate], the probability of transmission from host to vector (*β*) [which was not temperature-dependent in Gubbins et al. [6]] and the ratios of vectors to hosts (*m_C_* and *m_S_*). For the two-host, two-vector system, we propose to focus on the effects on *R*_0_ of varying the ratios of vectors to hosts (*m_C_*_1_, *m_C_*_2_, *m_S_*_1_, *m_S_*_2_) [linked to vector abundance], the probabilities of transmission from host to vector (*β*_1_, *β*_2_) [linked to vector competence and temperature-dependent in our model] and the vector host preferences (*σ*_1_, *σ*_2_). Our aim is to provide a general framework for two-host, two-vector approaches to bluetongue; however there is a paucity of data. There is one situation, South Africa, where *C. imicola* and *C. bolitinos* coexist across most of the country and for which we do have data. We undertake the analysis with reference to this.

As shown by Meiswinkel et al. [13], there are many areas (e.g. Western Cape, western part of the Eastern Cape, Mpumalanga, Gauteng and Limpopo Province) where *C. imicola* is 10 to 100 times more abundant than *C. bolitinos*. However, there are areas, in particular in the cooler high-lying areas of the Free State, where *C. bolitinos* is approximately 10 times more abundant than *C. imicola* [14] and Venter et al. [19] suggest that *C. bolitinos* may play an important role in the transmission of BTV in these areas. Paweska et al. [15] demonstrate that, regardless of incubation temperature (10, 15, 18, 23.5 or 30°C), the mean virus titre/midge, infection rate and proportion of infected females with transmission potential (i.e. virus titre/midge ≥10^3^ TCID_50_, where TCID_50_ (tissue culture infectious dose 50) is the amount of virus that will infect 50% of midges inoculated with it) are significantly higher in *C. bolitinos* than in *C. imicola* and suggest that, because of its significantly higher vector competence, *C. bolitinos* could be the primary vector in areas where it occurs in *lower* numbers than *C. imicola*, as well as in these cooler regions. Here, abundance is expressed through the ratios of vectors to hosts (*m_C_*_1_, *m_C_*_2_, *m_S_*_1_, *m_S_*_2_), while vector competence is expressed through the probabilities of transmission from host to vector (*β*_1_, *β*_2_). Regarding vector host preferences, there is evidence [14], [16] that many *Culicoides* species prefer to feed on cattle and some suggestion that *C. bolitinos* may not feed on sheep at all [16], [17].

### Estimating *β*_1_ and *β*_2_

Fu et al. [20] show that only midges containing ≥10^3^ TCID_50_ release detectable amounts of virus in their saliva. So, first we define ‘infectious’ as ‘having a virus titre ≥10^3^ TCID_50_’. Next we obtain from Table 2 of Paweska et al. [15], for several different temperatures, the proportion of vectors remaining that are infectious [i.e. (number of infectious vectors)/(number of initially susceptible vectors known to have fed on infected blood and still be alive after the incubation period)]. Each of these data points is equal to *β_j_* at a given temperature. By fitting curves to the data, we can find temperature-dependent functions for *β*_1_ (*C. imicola*) and *β*_2_ (*C. bolitinos*). Exponential curves of the form 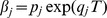 were fitted using a nonlinear least-squares method with bisquare weighting of the residuals. The coefficients and goodness of fit statistics are given in Table 2. The curves (shown in Appendix 3 in File S1) adequately describe the relationships between *β*_1_, *β*_2_ and temperature over this range of temperatures. Note that as temperature varies from 10 to 30°C, the ratio *β*_2_/*β*_1_ varies from 9.85 to 12.91 (i.e. the probability of transmission of *C. bolitinos* is always about 10 times greater than that of *C. imicola*).

**Table 2:** Coefficients and goodness of fit statistics for exponential curves of the form 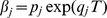, where *j* can be 1 (*C. imicola*) or 2 (*C. bolitinos*), fitted to data extracted from Paweska et al. [15].

	*C. imicola*	*C. bolitinos*
**Coefficients (with 95% confidence bounds)**		
*p*	0.0003699(−0.0002815, 0.001021)	0.005465(−0.0162, 0.02713)
*q*	0.1725(0.1111, 0.2339)	0.159(0.01987, 0.2982)
**Goodness of fit**		
sse	8.5345e-005	0.0519
adjrsquare	0.9648	0.8578
rmse	0.0046	0.1139

### Other parameter estimates

The estimates for *m_C_*_1_, *m_C_*_2_, *m_S_*_1_ and *m_S_*_2_ were based on catch sizes and species composition reported in Venter & Meiswinkel [14] and Venter et al. [17]. They are rough estimates designed to reflect the relative orders of magnitude of each vector species. As in Guis et al. [21], we assume that catch size approximates ratio of vectors to hosts. Estimates for the remaining parameters were taken from Gubbins et al. [6]. Details are given in Table 1. Six of these estimates *a_j_*, *ν_j_* and *μ_j_* (i.e. three for each vector species) depend on temperature. They are positive and increase monotonically for temperatures between 10.4°C and 35.5°C.

### Effect of differences in *m_ij_* and *β_j_*

In order to focus on the effects on *R*_0_ of differences in vector competence, vector abundance and vector host preference, the two species of vectors are assumed to be the same in every way except *β_j_*, *m_ij_* and *σ_j_*. We first consider the effect of differences in *m_ij_* for fixed *β_j_* and *σ_j_*.

**Figure 1:**
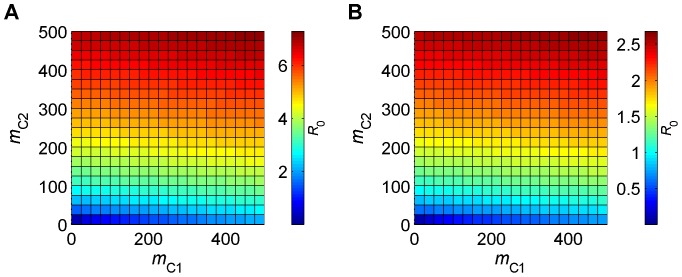
Effect on *R*_0_ of differences in the vector to host ratios *m*_C1_ and *m*_C2_. In (A) the temperature is 25°C, while in (B) it is 15°C. Parameter values (1 = *C. imicola*, 2 = *C. bolitinos*): *b*_1_ = 0.9, *b*_2_ = 0.9, *σ*_1_ = 0.5, *σ*_2_ = 0.5, *r*_C_ = 1/20.6, *r*_S_ = 1/16.4, *d*_C_ = 0, *d*_S_ = 0.005, *m*_S1_ = *m*_C1_, *m*_S2_ = *m*_C2_, *β*_1_, *β*_2_, *a*_1_, *a*_2_, *μ*_1_, *μ*_2_, *ν*_1_ and *ν*_2_ are determined by temperature.

**Figure 2:**
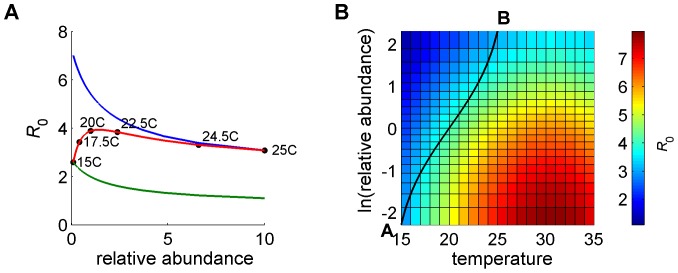
Effect on *R*_0_ of relative abundance and temperature. In (A) *R*_0_ is plotted against relative abundance (*m*_C1_/*m*_C2_ = *N*_1_/*N*_2_), which varies from 0.1 (when *C. bolitinos* is 10 times more abundant than *C. imicola*) to 10 (when *C. imicola* is 10 times more abundant than *C. bolitinos*). Temperature is either fixed at 25°C or 15°C or varies from 15°C to 25°C as relative abundance varies from 0.1 to 10. In (B) *R*_0_ is plotted against ln(relative abundance) and temperature. Parameter values (1 = *C. imicola*, 2 = *C. bolitinos*): *b*_1_ = 0.9, *b*_2_ = 0.9, *σ*_1_ = 0.5, *σ*_2_ = 0.5, *r*_C_ = 1/20.6, *r*_S_ = 1/16.4, *d*_C_ = 0, *d*_S_ = 0.005, *m*_S1_ = *m*_C1_, *m*_S2_ = *m*_C2_, *a*_1_, *a*_2_, *μ*_1_, *μ*_2_, *ν*_1_, *ν*_2_, *β*_1_ and *β*_2_ are determined by temperature.

First note that, as shown in equation (3), *φ*_C*j*_ varies with *m*_C*j*_, *m*_S*j*_ and *σ_j_*. However, when *m*_C*j*_ equals *m*_S*j*_, *φ*_C*j*_ (and hence *φ_j_*) depends on *σ_j_* alone. In Figure 1 (and Figure 2 below), *m*_S1_ and *m*_S2_ equal *m*_C1_ and *m*_C2_ respectively and *σ*_1_ = *σ*_2_ = 0.5. Consequently *φ*_1_ and *φ*_2_ are fixed at 0.67. The transmission probabilities *β*_1_ and *β*_2_ are determined (as described in Table 1) by temperature, which is 25°C in Figure 1A and 15°C in Figure 1B. The parameters *m*_C1_ and *m*_C2_ vary independently along the *x* and *y* axes respectively. In Figure 1A, we can clearly see that *R*_0_ is greater when vector 2 (*C. bolitinos*) is more abundant than vector 1 (*C. imicola*) [i.e. when *m*_C2_ is greater than *m*_C1_] than when the reverse is true. For example, when *m*_C1_ = 50 and *m*_C2_ = 500 (i.e. in the top left-hand corner), *R*_0_ is 7.0, whereas, when *m*_C1_ = 500 and *m*_C2_ = 50 (i.e. in the bottom right-hand corner), *R*_0_ is only 3.1. This large difference is due to the fact that *β*_2_ is approximately 10 times greater than *β*_1_ and illustrates the balance between vector abundance and vector competence for *C. imicola* and *C. bolitinos*. The same relationship is observed when the temperature is 15°C (i.e. in Figure 1B), but *R*_0_ is much smaller at this temperature.

It is also clear from Figure 1 that omitting one vector species (i.e. being constrained to one axis) leads to underestimation of *R*_0_ and when that species has a significantly higher vector competence (as does vector species 2) the degree of underestimation can be dramatic.

In Figure 2, *β*_1_ and *β*_2_ vary with temperature, as described in Table 1. The vector to host ratios *m_S_*_1_ and *m_S_*_2_ equal *m_C_*_1_ and *m_C_*_2_ respectively, which vary simultaneously with *x* as described below:
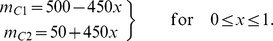
Relative abundance (*m*_C1_/*m*_C2_ = *N*_1_/*N*_2_) is therefore described by the hyperbola
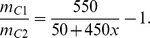



Figure 2A shows how *R*_0_ varies with relative abundance. Curves 1 (green) and 2 (blue) show the relationship when temperature is fixed at 15°C and 25°C, respectively. Curve 3 (red) is produced when temperature (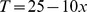 for 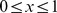) and relative abundance vary simultaneously with *x*. Hence, curve 3 (red) shows the relationship when temperature varies from 15°C to 25°C as relative abundance varies from 0.1 (when *C. bolitinos* is 10 times more abundant than *C. imicola*) to 10 (when *C. imicola* is 10 times more abundant than *C. bolitinos*). By definition, curve 3 is constrained to start at the same point as curve 1 and end at the same point as curve 2. Curve 3 can be thought of as a path across the landscape, moving from the cooler high-lying regions where *C. bolitinos* dominates to the warmer low-lying regions where *C. imicola* dominates. Along this path, temperature (and hence *β_j_*) and relative abundance vary simultaneously.

Figure 2B shows how *R*_0_ varies with relative abundance (y axis) and temperature (x axis) separately. It clearly shows that, for a fixed temperature, *R*_0_ always decreases as relative abundance of *C. imicola* increases. It also shows that, for fixed relative abundance, *R*_0_ initially increases with temperature, but starts to decrease again beyond 31°C. Curve 3 in Figure 2A corresponds to moving from A to B across the surface in Figure 2B. Along this path, the highest *R*_0_ corresponds to a relative abundance of approximately 1.4 and a temperature of approximately 21.1°C. In this case, for temperatures greater than 21.1°C, *R*_0_ drops with rising temperature because, at the same time, the less competent vector is replacing the more competent one.

In summary, we find that high vector competence can compensate for low vector abundance and that temperature, which determines the transmission probabilities *β*_1_ and *β*_2_ and also influences the abundance and composition of vector species, has a marked effect on *R*_0_.

### Effect of differences in *σ_j_*

**Figure 3:**
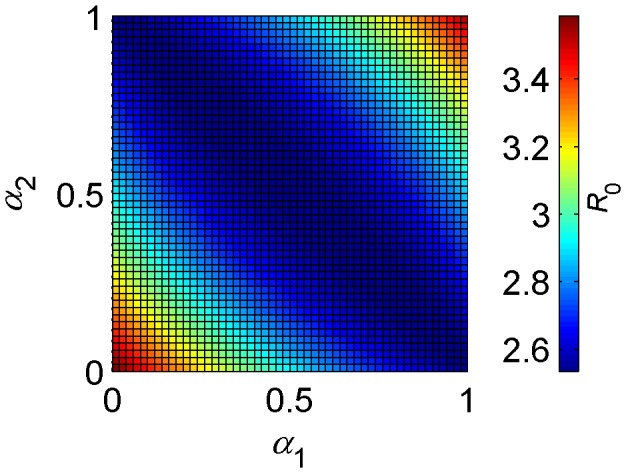
Effect on *R*_0_ of differences in the vector host preferences *α*_1_ and *α*_2_. Parameter values (1 = *C. imicola*, 2 = *C. bolitinos*): *b*_1_ = *b*_2_ = 0.9, *m*_C1_ = *m*_C2_ = *m*_S1_ = *m*_S2_ = 500, *r*_C_ = *r*_S_ = 1/16.4, *d*_C_ = *d*_S_ = 0.005, *a*_1_, *a*_2_, *μ*_1_, *μ*_2_, *ν*_1_, *ν*_2_ and *β*_1_ are determined by temperature *T*, where *T* = 25°C, *β*_2_ = *β*_1_.

We now consider the important effect that vector host preference (*σ_j_*) has on *R*_0_. In order to focus on the effect of *σ_j_*, we assume that the vector species differ only in *σ_j_*. Also, to ensure that there is no advantage to choosing cattle over sheep (or vice versa), we set *r*_C_ = *r*_S_, *d*_C_ = *d*_S_ and *m*_C1_ = *m*_S1_ = *m*_C2_ = *m*_S2_. When *σ_j_* = 0, the proportion of vectors of type *j* attracted to cattle (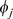) equals 1. When *σ_j_* = 1 (i.e. no preference), 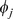 just depends on the relative numbers of each host species, with a greater number resulting in a greater share of the vectors. As *σ_j_*→∞, 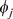
→0 where all vectors are attracted to sheep. To prevent loss of detail as *σ_j_*→∞, in Figure 3 we use *α_j_* rather than *σ_j_*, where *α_j_* is an alternative measure of vector host preference such that 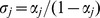. From this formula we can see that as *α_j_* varies from 0 to 1, *σ_j_* varies from 0 to ∞ and that *α_j_* = 0.5 indicates no preference.

Figure 3 clearly shows that the minimum value of *R*_0_ lies on a straight line running from (*α_1_* = 0, *α_2_* = 1), where 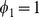 and 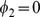 (i.e. vector type 1 feeds exclusively on cattle while vector type 2 feeds exclusively on sheep), through (*α_1_* = 0.5, *α_2_* = 0.5), where 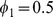 and 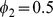 (i.e. neither vector has a preference and so both vector species are equally distributed between both host species), to (*α_1_* = 1, *α_2_* = 0), where 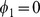 and 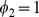 (i.e. vector 1 feeds exclusively on sheep while vector 2 feeds exclusively on cattle). Any deviation from this line results in a higher *R*_0_. This figure clearly shows two things: firstly, that different combinations of vector host preferences can result in the same *R*_0_; second, that when both vectors prefer the same host species, *R*_0_ is greater. This result is important because it shows that, when the vector species differ only in host preference and the host species are equally good as hosts (in this case, they share the same infectious period and the same pathogen-induced mortality rate) and equally abundant, overlooking vector host preference can result in an underestimation of *R*_0_.

In Figures 1 and 2 and Table 3, we used 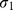 = 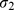 = 0.5 (which corresponds to 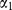 = 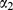 = 1/3) as many *Culicoides* species prefer to feed on cattle [14], [16]. However, while it is clear that *C. imicola* also feeds on sheep (and even horses and pigs too), there is some evidence that *C. bolitinos* does not – instead feeding exclusively on cattle and horses [16], [17]. A strong association between *C. bolitinos* and cattle is further suggested by the fact that *C. bolitinos* breeds in cattle dung [14], rather than soil like *C. imicola*. In terms of *R*_0_, if *C. bolitinos* does not feed on sheep, then 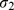 = 0 (i.e. 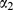 = 0) and the true value of *R*_0_ will be higher than our estimates based on 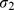 = 0.5.

**Table 3:** 2-host, 2-vector *R*_0_ and possible approximations based on the 2-host, 1-vector formula.

**Symbol**	**Description**	† **Formula in terms of ** ***R*** **_11_ and ** ***R*** **_22_**	***T*** ** = 25^o^C**		***T*** ** = 15^o^C**	
			**a**	**b**	**c**	**d**
			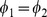	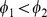	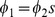	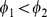
*R*_0_		Equation (1)	3.0736	3.4271	2.5860	3.5539
*R*_0,sum_	No cross-infection	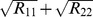	4.3464	4.9829	2.8098	3.7631
*R*_0,minβ_	Total *m* with min *β* and 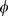	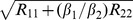	2.2492	2.0425	0.7776	0.7061
*R*_0,wtsum_	Weighted sum of *R*_0,minβ_ and *R*_0,maxβ_	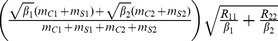	2.7086	2.7721	2.5261	3.4492
*R*_0,ave_	Total m with mean *β* and 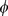	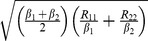	5.4032	5.8104	1.9875	2.1372
*R*_0,maxβ_	Total *m* with max *β* and 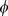	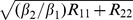	7.3028	10.0674	2.7010	3.7235

Parameter values (1 = *C. imicola*, 2 = *C. bolitinos*): *b*_1_ = 0.9, *b*_2_ = 0.9, *r*_C_ = 1/20.6, *r*_S_ = 1/16.4, *d*_C_ = 0, *d*_S_ = 0.005, *m*_S1_ = *m*_C1_, *m*_S2_ = *m*_C2_, *β*_1_, *β*_2_, *a*_1_, *a*_2_, *μ*_1_, *μ*_2_, *ν*_1_ and *ν*_2_ are determined by temperature, (a) *m*_C1_ = 500, *m*_C2_ = 50, *T* = 25°C, *σ*_1_ = 0.5 and *σ*_2_ = 0.5, (b) *m*_C1_ = 500, *m*_C2_ = 50, *T* = 25°C, *σ*_1_ = 1 and *σ*_2_ = 0, (c) *m*_C1_ = 50, *m*_C2_ = 500, *T* = 15°C, *σ*_1_ = 0.5 and *σ*_2_ = 0.5, (d) *m*_C1_ = 50, *m*_C2_ = 500, *T* = 15°C, *σ*_1_ = 1 and *σ*_2_ = 0.

† For the approximations *R*_0,minβ_, *R*_0,wtsum_, *R*_0,ave_ and *R*_0,maxβ_, the formula given is for 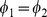 only. There is insufficient space to give the more general expressions.

### *R*_0_ approximations

The need for the two-host, two-vector formula is further emphasised when we consider several approximations based on the two-host, one-vector formula for *R*_0_, which is
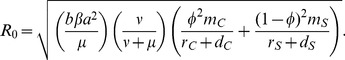
(4)


Suppose we have information on two vectors that are circulating in the same area and feeding on the same host populations. We might think it reasonable to assume that the vectors are acting independently, merely feeding on the same hosts. In which case, one option would be to calculate *R*_0_ for each vector separately and add them together. We refer to this approximation as *R*_0,sum_ (i.e. 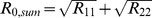). It incorporates the idea that there is no cross-infection. Table 3 contains the true value of *R*_0_ (i.e. calculated using the two host, two vector formula) and the value of *R*_0,sum_ under different scenarios. In examples a and b the temperature is 25°C and *C. imicola* is 10 times more abundant than *C. bolitinos* (representing warmer low-lying areas), whereas in examples c and d the temperature is 15°C and *C. bolitinos* is 10 times more abundant than *C. imicola* (representing cooler high-lying areas). In a and c, the vectors are assumed to have the same preference for cattle (i.e. *σ*_1_ = *σ*_2_ = 0.5) so 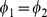. In b and d, *C. imicola* is assumed to have no preference for a particular host, while *C. bolitinos* is assumed to feed exclusively on cattle (i.e. *σ*_1_ = 1, *σ*_2_ = 0) so 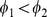. In these examples, *R*_0,sum_ consistently overestimates *R*_0_ by between 5% and 45%.

An alternative approach would be to pool the vectors (e.g. 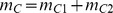 and 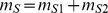) and use average values (e.g. 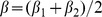 and 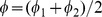). In the examples in Table 3, we have assumed that the vectors are very similar and so share many parameter values. In fact, we have assumed that they differ only in vector to host ratio (*m_ij_*), host to vector transmission probability (*β_j_*) and vector host preference (*σ_j_*). So, *R*_0,ave_ can be obtained by substituting 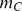, 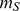, 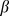 and 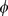 into equation (4). Surprisingly, the examples reveal that *R*_0,ave_ can sometimes overestimate and sometimes underestimate the true value by a significant amount.

Another possible approximation is obtained by first calculating the lower and upper bounds given by *R*_0,minβ_ and *R*_0,maxβ_ and then taking the weighted average (*R*_0,wtsum_). *R*_0,minβ_ is calculated in the same way as *R*_0,ave_ except that the host to vector transmission probability (*β*) takes the minimum value (*β*_1_, where 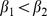), rather than the average, and the proportion of vectors attracted to cattle (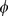) takes the minimum value (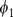, where 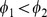), rather than the average. *R*_0,maxβ_ is the equivalent calculation using the maximum host to vector transmission probability (in this case *β*_2_) and the maximum proportion of vectors attracted to cattle (in this case 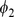) . *R*_0,wtsum_ is then the weighted sum of *R*_0,minβ_ and *R*_0,maxβ_ (i.e. 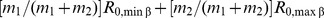, where 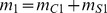 and 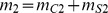). We can see from Table 3 that *R*_0,wtsum_ can provide a fairly good approximation to *R*_0_. In our examples, it consistently underestimates *R*_0_, but never by more than 19% and sometimes by as little as 2%. Alternative formulations in terms of *R*_11_ and *R*_22_ are given in Table 3 for comparison with *R*_0,sum_. Note however that, for *R*_0,ave_, *R*_0,minβ_, *R*_0,maxβ_ and *R*_0,wtsum_, the formula given is for 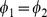 only. There is insufficient space to give the more general expression.

These examples suggest that, even when the vectors are very similar and share many parameter values, simply summing the contribution from each vector species (*R*_0,sum_) will lead to overestimation of *R*_0_ and that the degree of overestimation can be large. *R*_0,wtsum_ appears to provide a more consistent estimate. Table 3 also shows that intuitive approximations like *R*_0,ave_ can be very misleading, sometimes underestimating and sometimes overestimating the true value of *R*_0_.

## Discussion

We have presented an expression for *R*_0_ for a two-host, two-vector system and demonstrated its sensitivity to parameters relating to vector abundance, vector competence and vector host preference. We have shown that high vector competence can offset low vector abundance and that, where high vector competence and high vector abundance coincide, *R*_0_ can reach high values. We have also shown that the highest value of *R*_0_ does not always coincide with the highest *β_j_* values. Earlier work using a one-host, one-vector formulation showed that when *a*, *μ* and *ν* vary with temperature, *R*_0_ at first increases with temperature then decreases [22]. We observed the same behaviour when using the slightly different temperature-dependent functions described in Table 1. Figure 2B shows that this relationship is maintained when *β*_1_ and *β*_2_ also increase with temperature.

As shown in Figure 3, vector host preference has an interesting effect on *R*_0_. When the vector species differ only in host preference and the host species are equally good as hosts and equally abundant, a preference for one host species can increase *R*_0_ if the total feeding rate is maintained. When *both* vectors prefer the same host species, *R*_0_ *will* increase. When the preferred host benefits transmission (e.g. by having a longer infectious period, like cattle with bluetongue), then *R*_0_ will increase further. However, if the preferred host is less beneficial or more abundant, then *R*_0_ will decrease.

In this model, the vector species do not directly interact. They merely feed upon the same pool of susceptible hosts. So, we might expect a simpler formulation expressed in terms of the two-host, one-vector *R*_0_ for each species to provide a good approximation to *R*_0_. We considered several possibilities and found that simply summing the contribution from each vector species (*R*_0,sum_) leads to overestimation of *R*_0_, while using average values (*R*_0,ave_) can lead to under or overestimation. A more consistent estimate was provided by *R*_0,wtsum_. However, this approximation relies on the fact that the vectors differ in *m_ij_*, *β_j_* and *σ_j_* only. When the vectors differ in many ways, we can see from equation (1) that the two-host, one-vector formula will provide a good approximation when the level of cross-infection between vector species is very small. As this approaches the level of intraspecies infection, a combination of the two-host, one-vector *R*_0_ for each vector species (i.e. 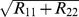) becomes a better estimate. Otherwise, particularly when the level of cross-infection is high, the two-host, two-vector formula is required for accurate estimation of *R*_0_.

The results of this work demonstrate the need for a two-host, two-vector formula for *R*_0_ in areas that support two significant vectors, particularly where those vectors differ in many ways. Further extensions of this model would be required for areas where there were more than two important vectors. Northern Europe could be one such area. Both *C. pulicaris* and *C. obsoletus* transmit bluetongue is this region. However, both of these vectors are in fact vector species groups containing multiple vector species (e.g. the *C. obsoletus* group contains four distinct vector species). At the moment, there is insufficient information about differences in vector competence between these species to be able to use this *R*_0_ formula (or an extension of it) in this region.

## Supporting Information

File S1 **Supporting information.**
(DOC)

## References

[1] Diekmann O, Heesterbeek JAP, Roberts MG (2010) The construction of next-generation matrices for compartmental epidemic models. J R Soc Interface 7: 873–885. 19892718 10.1098/rsif.2009.0386 PMC2871801

[2] Macdonald G (1955) The measurement of malaria transmission. Proc R Soc Med 48: 295–302. 14371594 10.1177/003591575504800409 PMC1918775

[3] Rogers DJ (1988) A general model for the African trypanosomiases. Parasitol 97: 193–212. 10.1017/s0031182000066853 3174235

[4] Lord CC, Woolhouse MEJ, Heesterbeek JAP, Mellor PS (1996) Vector-borne diseases and the basic reproduction number: a case study of African horse sickness. Med Vet Entomol 10: 19–28. 8834738 10.1111/j.1365-2915.1996.tb00077.x

[5] Hartemink NA, Purse BV, Meiswinkel R, Brown HE, de Koeijer A, et al (2009) Mapping the basic reproduction number (*R* _0_) for vector-borne diseases: A case study on bluetongue virus. Epidemics 1: 153–161. 21352762 10.1016/j.epidem.2009.05.004

[6] Gubbins S, Carpenter S, Baylis M, Wood JLN, Mellor PS (2008) Assessing the risk of bluetongue to UK livestock: uncertainty and sensitivity analyses of a temperature-dependent model for the basic reproduction number. J R Soc Interface 5: 363–371. 17638649 10.1098/rsif.2007.1110 PMC2497440

[7] Lopez LF, Coutinho FAB, Burattini MN, Massad E (2002) Threshold conditions for infection persistence in complex host-vectors interactions. C R Biologies 325: 1073–1084. 12506721 10.1016/s1631-0691(02)01534-2

[8] Pessanha JEM, Caiaffa WT, Cecilio AB, Iani FC de M, Araujo SC, et al (2011) Cocirculation of two dengue virus serotypes in individual and pooled samples of *Aedes aegypti* and *Aedes albopictus* larvae. Rev Soc Bras Med Trop 44: 103–105. 21340419 10.1590/s0037-86822011000100023

[9] Suryanarayana Murty U, Srinivasa Rao M, Arunachalam N (2010) The effects of climatic factors on the distribution and abundance of Japanese encephalitis vectors in Kurnool district of Andhra Pradesh, India. J Vector Borne Dis 47: 26–32. 20231770

[10] Tanga MC, Ngundu WI, Tchouassi PD (2011) Daily survival and human blood index of major malaria vectors associated with oil palm cultivation in Cameroon and their role in malaria transmission. Trop Med Int Health 16: 447–457. 21244587 10.1111/j.1365-3156.2011.02726.x

[11] Purse BV, Mellor PS, Rogers DJ, Samuel AR, Mertens PPC, et al (2005) Climate change and the recent emergence of bluetongue in Europe. Nat Rev Microbiol 3: 171–181. 15685226 10.1038/nrmicro1090

[12] Torina A, Caracappa S, Mellor PS, Baylis M, Purse BV (2004) Spatial distribution of bluetongue virus and its *Culicoides* vectors in Sicily. Med Vet Entomol 18: 81–89. 15189232 10.1111/j.0269-283X.2004.00493.x

[13] Meiswinkel R, Labuschagne K, Baylis M, Mellor PS (2004) Multiple vectors and their differing ecologies: observations on two bluetongue and African horse sickness vector *Culicoides* species in South Africa. Vet Ital 40: 296–302. 20419682

[14] Venter GJ, Meiswinkel R (1994) The virtual absence of *Culicoides imicola* (Diptera: Ceratopogonidae) in a light-trap survey of the colder, high-lying area of the eastern Orange Free State, South Africa, and implications for the transmission of arboviruses. Onderstepoort J Vet 61: 327–340. 7501364

[15] Paweska JT, Venter GJ, Mellor PS (2002) Vector competence of South African *Culicoides* species for bluetongue virus serotype 1 (BTV-1) with special reference to the effect of temperature on the rate of virus replication in *C. imicola* and *C. bolitinos* . Med Vet Entomol 16: 10–21. 11963973 10.1046/j.1365-2915.2002.00334.x

[16] Venter GJ, Meiswinkel R, Nevill EM, Edwardes M (1996) *Culicoides* (Diptera: Ceratopogonidae) associated with livestock in the Onderstepoort area, Gauteng, South Africa as determined by light-trap collections. Onderstepoort J Vet 63: 315–325. 9173363

[17] Venter GJ, Nevill EM, van der Linde TCde K (1996) Geographical distribution and relative abundance of stock-associated *Culicoides* (Diptera: Ceratopogonidae) in southern Africa in relation to their potential as viral vectors. Onderstepoort J Vet 63: 25–38. 8848300

[18] Garros C, Gardes L, Allene X, Rakotoarivony I, Viennet E, et al (2011) Adaptation of a species-specific multiplex PCR assay for the identification of blood meal source in *Culicoides* (Ceratopogonidae: Diptera): applications on Palaearctic biting midge species, vectors of Orbiviruses. Infection, Genetics and Evolution 11: 1103–1110. 10.1016/j.meegid.2011.04.002 21511056

[19] Venter GJ, Paweska JT, van Dijk AA, Mellor PS, Tabachnick WJ (1998) Vector competence of *Culicoides bolitinos* and *C. imicola* (Diptera: Ceratopogonidae) for South African bluetongue virus serotypes 1, 3 and 4. Med Vet Entomol 12: 101–108. 10.1046/j.1365-2915.1998.00116.x 9824821

[20] Fu H, Leake CJ, Mertens PPC, Mellor PS (1999) The barriers to bluetongue virus infection, dissemination and transmission in the vector, *Culicoides variipennis* (Diptera: Ceratopogonidae). Arch Virol 144: 747–761. 10365165 10.1007/s007050050540

[21] Guis H, Caminade C, Calvete C, Morse AP, Tran A, et al (2011) Modelling the effects of past and future climate on the risk of bluetongue emergence in Europe. J R Soc Interface DOI: 10.1098/rsif.2011.0255. 10.1098/rsif.2011.0255 PMC3243388 21697167

[22] de Koeijer AA, Elbers ARW (2007) Modelling of vector-borne diseases and transmission of bluetongue virus in North-West Europe. In: Takken W, Knols BGJ, editors. Emerging pests and vector-borne diseases in Europe - Ecology and control of vector-borne diseases vol. 1. The Netherlands: Wageningen Academic Publishers. pp. 99–112.

[23] Nevill EM (1978) The use of cattle to protect sheep from bluetongue infection. J S Afr Vet Assoc 49: 129–130. 215768

