# Supplementary material for: Two-Host, Two-Vector Basic Reproduction Ratio (R 0) for Bluetongue
Source: PLoS One. 2013 Jan 8;8(1):e53128. doi: 10.1371/journal.pone.0053128 (PMC3540086; doi:10.1371/journal.pone.0053128)
Supplement: File S1 — Supporting information. (DOC) [file pone.0053128.s001.doc]

**Supporting Information**

**Two-host, two-vector basic reproduction ratio (*R*_0_) for bluetongue**

Joanne Turner, Roger G. Bowers and Matthew Baylis

**Appendix 1: Derivation of *R*_0_**

Following the method described by van den Driessche & Watmough [S1], we calculate *R*_0_ by separating new infections from all other changes in the population. Let *x* be a vector containing the number of individuals in each compartment. Let $\Psi_{p}(x)$ be the rate at which new infections appear in compartment *p* and $\Phi_{p}(x)$ be the rate at which individuals leave (or, if the rate is negative, are added to) compartment *p* by any other means. For the model described in the main text,

$\Psi=\left(\begin{matrix} (\lambda_{HC})X^{C} \\ (\lambda_{HS})X^{S} \\ \lambda_{V1}S_{1} \\ \lambda_{V2}S_{2} \\ 0 \\ 0 \end{matrix}\right)$ $\Phi=\left(\begin{matrix} (r_{C}+d_{C})Y^{C} \\ (r_{S}+d_{S})Y^{S} \\ (v_{1}+\mu_{1})L_{1} \\ (v_{2}+\mu_{2})L_{2} \\ -v_{1}L_{1}+\mu_{1}I_{1} \\ -v_{2}L_{2}+\mu_{2}I_{2} \end{matrix}\right)$.

Note that there are six compartments: (1) infectious cattle, *Y^C^* ; (2) infectious sheep, *Y^S^* ; (3) latent vectors of type 1, *L_1_* ; (4) latent vectors of type 2, *L_2_* ; (5) infectious vectors of type 1, *I_1_*, and (6) infectious vectors of type 2, *I_2_*. As shown in [S1], *R*_0_ is the dominant eigenvalue of the next-generation matrix *K*, which is given by $K=FV^{-1}$ where

$F=\left[\frac{\partial\Psi_{p}}{\partial x_{q}}(x_{0})\right]$, $V=\left[\frac{\partial\Phi_{p}}{\partial x_{q}}(x_{0})\right]$, $1\leq p,q\leq6$.

The partial derivatives are evaluated at the disease-free equilibrium denoted by *x*_0_. It follows that

$K=\left(\begin{matrix} 0 & 0 & k_{13} & k_{14} & k_{15} & k_{16} \\ 0 & 0 & k_{23} & k_{24} & k_{25} & k_{26} \\ k_{31} & k_{32} & 0 & 0 & 0 & 0 \\ k_{41} & k_{42} & 0 & 0 & 0 & 0 \\ 0 & 0 & 0 & 0 & 0 & 0 \\ 0 & 0 & 0 & 0 & 0 & 0 \end{matrix}\right)$,

where $k_{pq}$ is the number of new cases in compartment *p* produced by an infectious individual from compartment *q*. The elements of *K* are given in Table S1. Note that $\varphi_{j}$ and $1-\varphi_{j}$ have replaced $\varphi_{Cj}$ and $\varphi_{Sj}$ respectively.

Table S1: Elements of next generation matrix *K*.

| **Elements for vector species 1** | **Formula** | **Elements for vector species 2** | **Formula** |
| --- | --- | --- | --- |
| $k_{13}$ | $\frac{b_{1}a_{1}\varphi_{1}\nu_{1}}{\mu_{1}(\nu_{1}+\mu_{1})}$ | $k_{14}$ | $\frac{b_{2}a_{2}\varphi_{2}\nu_{2}}{\mu_{2}(\nu_{2}+\mu_{2})}$ |
| $k_{15}$ | $\frac{b_{1}a_{1}\varphi_{1}}{\mu_{1}}$ | $k_{16}$ | $\frac{b_{2}a_{2}\varphi_{2}}{\mu_{2}}$ |
| $k_{23}$ | $\frac{b_{1}a_{1}(1-\varphi_{1})\nu_{1}}{\mu_{1}(\nu_{1}+\mu_{1})}$ | $k_{24}$ | $\frac{b_{2}a_{2}(1-\varphi_{2})\nu_{2}}{\mu_{2}(\nu_{2}+\mu_{2})}$ |
| $k_{25}$ | $\frac{b_{1}a_{1}(1-\varphi_{1})}{\mu_{1}}$ | $k_{26}$ | $\frac{b_{2}a_{2}(1-\varphi_{2})}{\mu_{2}}$ |
| $k_{31}$ | $\frac{\beta_{1}a_{1}\varphi_{1}m_{C1}}{r_{C}+d_{C}}$ | $k_{41}$ | $\frac{\beta_{2}a_{2}\varphi_{2}m_{C2}}{r_{C}+d_{C}}$ |
| $k_{32}$ | $\frac{\beta_{1}a_{1}(1-\varphi_{1})m_{S1}}{r_{S}+d_{S}}$ | $k_{42}$ | $\frac{\beta_{2}a_{2}(1-\varphi_{2})m_{S2}}{r_{S}+d_{S}}$ |

From $\left|K-\lambda I\right|=0$, we find that *R*_0_ is the largest solution of

$\lambda^{4}-\lambda^{2}(k_{31}k_{13}+k_{32}k_{23}+k_{41}k_{14}+k_{42}k_{24})+(k_{31}k_{42}-k_{32}k_{41})(k_{13}k_{24}-k_{14}k_{23})=0$. (A1.1)

Although finding the dominant eigenvalue of *K* gives the correct result, there are certain drawbacks to using this approach, namely the necessity of working with a 6x6 matrix and the difficulty in interpreting the result biologically. One alternative is to find the dominant eigenvalue of *BA* (a 4x4 submatrix of *K*^2^), where matrix *A* describes vector to host transmission and matrix *B* describes host to vector transmission. Not only is *BA* smaller than *K*, the elements have an obvious biological interpretation and, as shown in Appendix 2, *R*_0_ equals the square root of the dominant eigenvalue of *BA*.

For the model described in the main text,

$$K=\left(\begin{matrix} 0 & A \\ B & 0 \end{matrix}\right)$$

with

$A=\left(\begin{matrix} k_{13} & k_{14} & k_{15} & k_{16} \\ k_{23} & k_{24} & k_{25} & k_{26} \end{matrix}\right)$ and $B=\left(\begin{matrix} k_{31} & k_{32} \\ k_{41} & k_{42} \\ 0 & 0 \\ 0 & 0 \end{matrix}\right)$.

Therefore,

$BA=\left(\begin{matrix} k_{31}k_{13}+k_{32}k_{23} & k_{31}k_{14}+k_{32}k_{24} & k_{31}k_{15}+k_{32}k_{25} & k_{31}k_{16}+k_{32}k_{26} \\ k_{41}k_{13}+k_{42}k_{23} & k_{41}k_{14}+k_{42}k_{24} & k_{41}k_{15}+k_{42}k_{25} & k_{41}k_{16}+k_{42}k_{26} \\ 0 & 0 & 0 & 0 \\ 0 & 0 & 0 & 0 \end{matrix}\right)$.

Conveniently, this can be rewritten as

,

where $R_{ij}$ equals the average number of infectious vectors of type *i* produced by one infectious vector of type *j* (in two generations). The element $\tilde{R}_{ij}$ is similar to $R_{ij}$, but does not represent a complete cycle of infection. For example, $\tilde{R}_{11}$ is the average number of *latent* vectors of type 1 produced by one *infectious* vector of type 1 and as such does not take into account the extrinsic incubation period.

From $\left|BA-\eta I\right|=0$, we find that the dominant eigenvalue is the largest solution of

$\eta^{2}-\eta(R_{11}+R_{22})+(R_{11}R_{22}-R_{12}R_{21})=0$.

This is equivalent to equation (A1.1) with $\eta=\lambda^{2}$. It follows that

$R_{0}=\sqrt{\eta}=\lambda=\sqrt{\frac{1}{2}[(R_{11}+R_{22})+\sqrt{(R_{11}+R_{22})^{2}-4(R_{11}R_{22}-R_{12}R_{21})}]}$.

Note that $R_{11}+R_{22}\geq0$ always and $(R_{11}+R_{22})^{2}-4(R_{11}R_{22}-R_{12}R_{21})=(R_{11}-R_{22})^{2}+4R_{12}R_{21}\geq0$ always.

It is possible to derive *R*_0_ by finding the dominant eigenvalue of *AB*, which is a 2x2 matrix for any number of vector species. However, the interpretation is cast in terms of the average number of infectious hosts of type *i* produced by one infectious host of type *j* (in two generations). As such, it has a direct analogy with a one-host, two-vector formula for *R*_0_, rather than the two-host, one vector formula published by Gubbins et al. [6].

It is worth noting that, in contrast to directly-transmitted infections, for vector-borne infections $R_{0}^{2}$ makes more sense biologically [2] and is in fact what is measured in the field (i.e. two-generation ‘like’ to ‘like’ transmission).

**Appendix 2: Eigenvalues of *K* and *BA***

For completeness, we include proof that *R*_0_, the dominant eigenvalue of *K*, is the square root of the dominant eigenvalue of both *AB* and *BA*, where matrix *A* describes vector to host transmission and matrix *B* describes host to vector transmission.

With *A* and *B* defined as above, the one-step next generation matrix is

$$K=\left(\begin{matrix} 0 & A \\ B & 0 \end{matrix}\right)$$

and the two-step matrix is

$K^{2}=\left(\begin{matrix} 0 & A \\ B & 0 \end{matrix}\right)\left(\begin{matrix} 0 & A \\ B & 0 \end{matrix}\right)=\left(\begin{matrix} AB & 0 \\ 0 & BA \end{matrix}\right)$,

where *AB* describes host to vector to host transmission and *BA* describes vector to host to vector transmission. The eigenvalues of *K* satisfy

$\left(\begin{matrix} 0 & A \\ B & 0 \end{matrix}\right)\left(\begin{matrix} u \\ v \end{matrix}\right)=\lambda\left(\begin{matrix} u \\ v \end{matrix}\right)$,

where at least one of *u* and *v* is non-zero. This can be rewritten as

$\left(\begin{matrix} Av \\ Bu \end{matrix}\right)=\lambda\left(\begin{matrix} u \\ v \end{matrix}\right)$.

So, we find that

$\begin{matrix} Av=\lambda u \\ Bu=\lambda v \end{matrix}$ (A2.1)

It follows that

$\begin{matrix} ABu=\lambda^{2}u \\ BAv=\lambda^{2}v \end{matrix}$ (A2.2)

From (A2.1), we see that when $\lambda\neq0$ and $u\neq0$, then $v\neq0$. This combined with (A2.2) reveals that any *non-zero* eigenvalue of *AB* is also an eigenvalue of *BA*. As the eigenvalues of *K*^2^ are the square of the eigenvalues of *K*, we can conclude from (A2.2) that any *non-zero* eigenvalue (including $R_{0}^{2}$) of the two-step matrix *K*^2^ is an eigenvalue of *both* *AB* and *BA*. For $\lambda=0$ to be an eigenvalue of *K*, at least one of *u* and *v* has to be non-zero. So, when $\lambda=0$, at least one of the equations in (A2.2) will be an eigenvalue equation. In other words, any *zero* eigenvalue of *K*^2^ is an eigenvalue of *at least one* of *AB* and *BA*.

More detail follows by adapting Abadir & Magnus [S2]. Let *A* be an *m* x *n* matrix and *B* an *n* x *m* matrix. Notice that the following equation is true.

$$\left(\begin{matrix} AB-\lambda I_{m} & -A \\ 0 & -\lambda I_{n} \end{matrix}\right)\left(\begin{matrix} I_{m} & 0 \\ B & I_{n} \end{matrix}\right)=\left(\begin{matrix} I_{m} & 0 \\ B & I_{n} \end{matrix}\right)\left(\begin{matrix} -\lambda I_{m} & -A \\ 0 & BA-\lambda I_{n} \end{matrix}\right)$$

By taking determinants on both sides we obtain

$$\left|\begin{matrix} AB-\lambda I_{m} & -A \\ 0 & -\lambda I_{n} \end{matrix}\right|=\left|\begin{matrix} -\lambda I_{m} & -A \\ 0 & BA-\lambda I_{n} \end{matrix}\right|$$

$$(-\lambda)^{n}\left|AB-\lambda I_{m}\right|=(-\lambda)^{m}\left|BA-\lambda I_{n}\right|$$

When $\lambda\neq0$,

$$\left|BA-\lambda I_{n}\right|=(-\lambda)^{n-m}\left|AB-\lambda I_{m}\right|$$

Hence, the non-zero eigenvalues of *BA* are the same as the non-zero eigenvalues of *AB*.

As *AB* has at most *m* non-zero eigenvalues and *BA* has *n* eigenvalues in total (assuming *n*>*m*), *BA* must also have at least *n*-*m* zero eigenvalues. In fact, if $\left|AB-\lambda I_{m}\right|=0$ has no zero roots, then *BA* has precisely *n*-*m* zero eigenvalues. If $\left|AB-\lambda I_{m}\right|=0$ has one or more zero roots, then *BA* has more than *n*-*m* zero eigenvalues.

**Appendix 3: Temperature-dependent *β* curves**


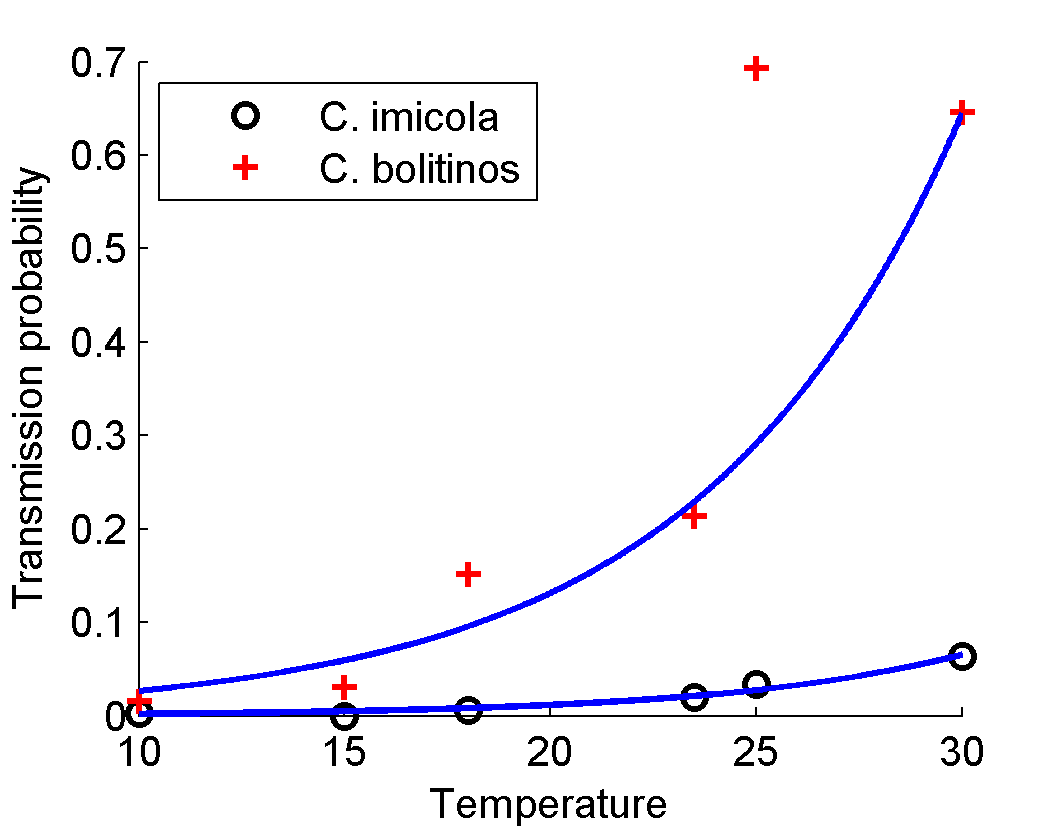


Figure S1: Relationship between the transmission probabilities *β*_1_ and *β*_2_ and temperature.

Temperature-dependent functions for *β*_1_ (*C*. *imicola*) and *β*_2_ (*C*. *bolitinos*) were found by fitting exponential curves of the form $\beta_{j}=p_{j}\exp(q_{j}T)$ to data from Paweska et al. [15]. A nonlinear least-squares method was used with bisquare weighting of the residuals. The coefficients and goodness of fit statistics are given in Table 2 in the main text. As shown in Figure S1, the curves adequately describe the relationships between *β*_1_, *β*_2_ and temperature over this range of temperatures.

**References**

S1. van den Driessche P, Watmough J (2002) Reproduction numbers and sub-threshold endemic equilibria for compartmental models of disease transmission. Math Biosci 180: 29-48.

S2. Abadir KM, Magnus JR (2005) Matrix Algebra. UK: Cambridge University Press. 434 p.
